# Teaching in the Digital Age—Developing a Support Program for Nursing Education Providers: Design-Based Research

**DOI:** 10.2196/66109

**Published:** 2025-01-15

**Authors:** Stefan Walzer, Carolin Barthel, Ronja Pazouki, Helga Marx, Sven Ziegler, Peter Koenig, Christiane Kugler, Stefan Jobst

**Affiliations:** 1 Care and Technology Lab Furtwangen University Furtwangen im Schwarzwald Germany; 2 Institute of Nursing Science Faculty of Medicine University of Freiburg Freiburg Germany; 3 Nursing Direction Medical Center University of Freiburg Freiburg Germany

**Keywords:** digital competencies, nursing education, support program, needs assessment, design-based research, feasibility study, nursing education provider, qualitative research, nurse, health care, focus group, digital age, expert consultation, thematic content analysis, feasibility test, satisfaction, competency-based approach, workplace barrier, health care digitalization, digital technology

## Abstract

**Background:**

Health care systems and the nursing profession worldwide are being transformed by technology and digitalization. Nurses acquire digital competence through their own experience in daily practice, but also from education and training; nursing education providers thus play an important role. While nursing education providers have some level of digital competence, there is a need for ongoing training and support for them to develop more advanced skills and effectively integrate technology into their teaching.

**Objective:**

This study aims to develop a needs-based support program for nursing education providers to foster digital competencies and to test this intervention.

**Methods:**

We used a design-based research approach, incorporating iterative development with expert consultation to create and evaluate a support program for nursing education providers. Focus groups were conducted online to assess needs, and thematic content analysis was used to derive key insights. The support program was then refined through expert feedback and subjected to a feasibility and satisfaction test, with participant evaluations analyzed descriptively.

**Results:**

Six main categories emerged from the focus groups, highlighting key areas, including the use of digital technology, ongoing support needs, and the current state of digitalization in nursing education. The support program was developed based on these findings, with expert validation leading to adjustments in timing, content prioritization, and platform integration. Preliminary testing showed good overall satisfaction with the support program, although participants suggested improvements in content relevance and digital platform usability.

**Conclusions:**

Although the feasibility test showed high satisfaction with the support program, low participation rates and limited perceived knowledge gain were major concerns. The results suggest that while the program was well received, further refinements, including a focus on competency-based approaches and addressing workplace barriers, are needed to increase participation and effectiveness of such interventions. The findings of this research can be used as a basis for the development of similar programs in other educational and health care contexts.

## Introduction

### Background

Technology and digitalization are transforming health care systems and the nursing profession globally [1]. The range of modern technologies used in health care practice is broad, with varying levels of complexity and includes, among others, electronic health records, sensors, tracking, artificial intelligence, and robotics [2,3]. Digital competence consists of knowledge, attitudes, and skills “required to use new technologies in a meaningful way and as a tool for learning, working and leisure time, understanding the essential phenomena of digital technologies in society as well as in one’s own life, and the motivation to participate in the digital world as an active and responsible actor” (pp. 670-671) [4].

Digital competence for nurses is acquired through their own experience in daily practice, but also in continuing education and training, where the providers of this education play an important role [5,6]. In Germany, nursing is an apprenticeship-based profession. Educational activities during this apprenticeship are performed by nurse educators, who provide theoretical education to nursing students, and clinical mentors, who provide supervision and mentoring of trainees, students, or new professionals; these roles are referred to together as “nursing education providers” in this paper. The key aspects of the training and the characteristics of these job designations are summarized in Multimedia Appendix 1. Nursing education providers are responsible for designing and delivering educational programs that include the use of digital technologies [7]. These responsibilities involve developing digital teaching materials and resources for use in the classroom or online, in addition to providing hands-on training and support to help students or new professionals develop their digital competencies [8]. There are already several digital technologies being used in theoretical and practical nursing education, for example, laptops, headphones, and video cameras for online or hybrid teaching, digital blackboards, simulation dolls, and digital platforms [9]. The use of these technologies requires that nursing education providers are capable of imparting the necessary competencies and that they are also able to use digital technologies themselves through the demonstration of pedagogical digital competence in their work [10].

Internationally, several studies have investigated the evidence regarding the digital competence of nursing education providers. A study by Forman et al [11] showed barriers against the implementation of technology in teaching, as well as the need for better support for nursing education providers who want to use technology in educational activities. Furthermore, they pointed out a missing consensus about the definition of minimum levels of digital competence [11]. Similarly, Männistö et al [8] described competence as a multifaceted phenomenon, which depends on various factors. It is proven by multiple studies that a lack of knowledge regarding technology among nursing education providers leads to worse outcomes in the preparation of nursing students [12].

A survey of 169 nursing education providers in a German medical center assessed their self-perceived competence in using digital technologies for teaching [13]. The results showed that nurses generally feel competent and positive about using digital technology in educational activities. While a basic setup of digital teaching tools is available, respondents expressed a need for further training in digital competence. The study’s quantitative approach provided insights into digital competence, but the authors recommend qualitative methods to deepen understanding and develop practical recommendations.

Overall, the evidence suggests that while nursing education providers have some level of digital competence, there is a need for ongoing training and support to help them develop more advanced skills and effectively incorporate technology into their teaching. Although the topic is attracting increasing international interest, there still seem to be no established training programs focused on digitalization in nursing education in several countries, including Germany [14].

### Aim

The aims of this study were (1) to develop a needs-based support program for nursing education providers to foster digital competencies in a specific context at a university hospital in Germany and (2) to test this program regarding feasibility and acceptance.

## Methods

### Overview

This research report was written according to the GUIDED (Guidance for Reporting Intervention Development Studies in Health Research) [15] and the TIDieR (Template for Intervention Description and Replication) checklists [16]. Both checklists can be found in Multimedia Appendices 2 and 3.

### Design

The design of this study was based on the design-based research methodology as described by Anderson and Shattuck [17]. Design-based research is an iterative approach that aims to improve educational practices through the design, implementation, and refinement of interventions in real-world settings [17]. This methodology emphasizes stakeholder collaboration, iterative refinement, and empirical testing, making it well suited to the complexities of developing and testing a support program for nursing education providers [17] and has already proven effective for digital innovation in education [18]. Within design-based research methodology, the development of the support program followed a step-by-step, iterative, and participatory process with the consultative involvement of experts of the target group, based on the recommendations for the development of educational programs by Schneiderhahn et al [19], Schlutz [20], and the Medical Research Council framework for the development and evaluation of complex interventions in health care [21]. Schlutz [20] emphasizes the importance of developing educational services that respond to the specific needs of learners within their professional contexts. His focus on participatory approaches helps to ensure that the support program we develop is not only relevant but also tailored to the challenges faced by nursing education providers in the digital age. Similarly, Schneiderhan et al [19] present a systematic guide to curriculum development that emphasizes the need to align educational objectives with learner needs and competencies. Their emphasis on stakeholder involvement and iterative revision is consistent with our design-based research methodology, enhancing the program’s effectiveness and ensuring its relevance to nursing education. The Medical Research Council framework [20] further contributes to our study by providing a structured approach to the development and evaluation of complex interventions. It ensures that our support program is contextually appropriate, and incorporates iterative feedback and refinement throughout the development process.

This study was conducted and coordinated by a group of nurse researchers with expertise in the fields of digitization (SW and SZ), adult education (CB and HM), and intervention development (SJ). One group member (SJ) was already involved in the previous quantitative survey mentioned above [13].

### Procedures

**Figure 1:**
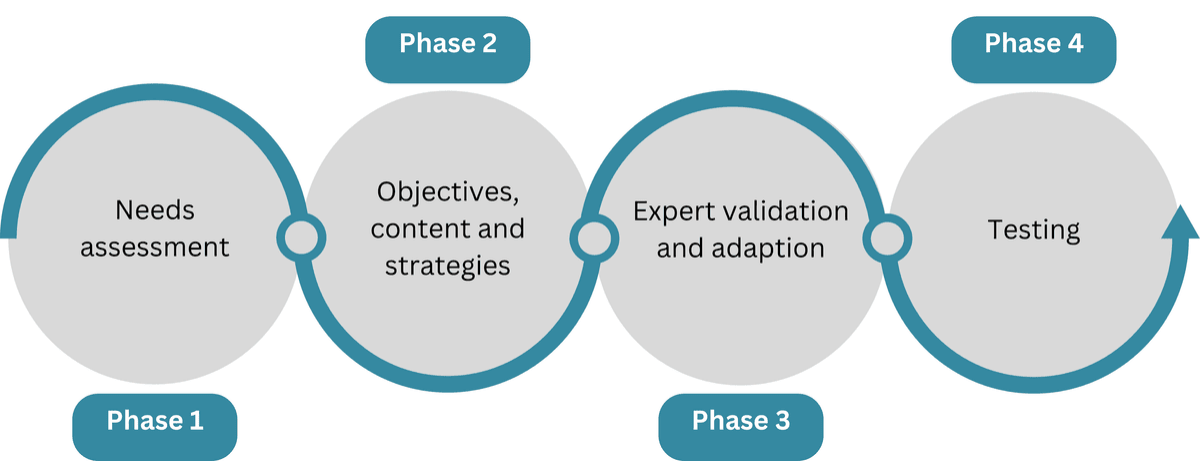
Support program development steps.

In the context of this study, the phases for curriculum development according to Schneiderhan et al [19] and Schlutz [20] were integrated into the process of design-based research for the detailed design of the learning situation. The phases and the iterative approach are illustrated in Figure 1 and are outlined in the following sections.

### Phase 1: Needs Assessment

#### Overview

Based on an explanatory sequential design [20], this phase adopted a qualitative research approach using focus groups to assess the needs of nursing education providers based on the results of the mentioned quantitative survey [13]. Focus groups are a special type of group interview where a moderator leads a discussion focused on a particular subject [22]. They are useful for obtaining information about beliefs, attitudes, thoughts, and perceptions on a specific topic from multiple individuals in a short amount of time. Participant interaction within the group plays a vital role in generating the data [22-24].

An interview guideline was developed by the research group following the recommendations of Kallio et al [25]. A semistructured interview guideline was chosen because it focused on the concepts of interest while still allowing flexibility. Sequential data integration [26] was performed by using the structure and content of the guideline as a base for the structure of the written survey questionnaire used in the study of Jobst et al [13] and the core findings to be explored in greater depth. During the development phase, preliminary versions of the questionnaire went through several internal reviews and adjustments by all members of the research team. The themes of the final guideline were as follows: (1) existence and use of digital technologies; (2) assessment of digital competencies; (3) support; and (4) age, gender, and specialty area. Each topic area was introduced in advance by a brief presentation of corresponding results from the written survey to provide context for participants and to encourage them to discuss their experiences related to the topic of interest. Due to hygiene regulations (caused by the COVID-19 pandemic) at the time the study was conducted, focus groups were scheduled as online sessions. For this purpose, the videoconferencing service WebEx (Cisco) was used which operated via the secure servers of the institution. The research team programmed technical support if necessary. Although the members of the research team and potential participants belong to the same professional group, there is no overlap between their respective fields of work. Thus, it was assumed that there is no dependency relationship or conflict of interest between the research team and the participants.

#### Participants

Nursing education providers were determined as the target group for participation in this study, as they had specific knowledge and experience related to the objectives under investigation. The teaching activity in nursing education can be considered as a common characteristic of the participants, which facilitates the creation of a pleasant atmosphere for discussion and exchange of opinions, as well as promoting interaction between them. A purposive sampling approach [27] was used to identify and recruit nursing education providers working at the above-mentioned medical center and the associated nursing school. Prerequisites for participation in the focus groups were membership in the professional group of nursing education providers employed at the University Medical Center, being aged at least 18 years, providing a signed consent form, and having to access to technology to use the videoconferencing service. Prior to starting the recruitment process, permission was obtained from the institution’s staff council, which approved the involvement of nursing education providers. Potential participants were contacted via email using internal institutional mailing lists and asked to respond in writing if they were interested in participating in the study. Interested individuals received detailed written information about the study and participation, as well as a written informed consent form.

#### Data Collection

The two focus groups were led by two of the authors (SW and SJ), of whom one already had experience in conducting interviews. They were assisted by two other members of the research team who took field notes or asked supplementary questions. The focus group discussions were audio recorded and transcribed using a denaturalized approach in which idiosyncratic speech elements were omitted [28]. Field notes written during the discussions were merged and converted into a digital text format. Transcripts and field notes were pseudonymized prior to analysis. The participants received a demographic questionnaire prior to the focus group discussion.

#### Analysis

The qualitative data in textual form collected from the transcribed focus group discussions were analyzed using MAXQDA 2022 (VERBI Software). Content analysis using a deductive-inductive approach using the method of thematic qualitative text analysis was performed [29]. To analyze the data, a deductive categorization matrix was developed with four main categories based on the themes of the interview guideline. Content was assigned to these main categories and respective subcategories were inductively formed in the process. Codes that did not fit into the categorization matrix were combined into inductively formed new main categories and subcategories during the analysis process [30]. Prior to the coding process, the categorization matrix was pilot-tested by two members of the research team to check its fit and feasibility. Six coders divided into three groups sequentially coded the transcripts, each group building on the interpretive work of the previous ones. First, initial codes were developed in a process of condensation, followed by an abstraction process, where codes were then combined into categories that were allocated to the main categories of the predefined categorization matrix. After the end of this phase, coders met to discuss the resulting codes and categories, their classification in the categorization matrix, and any discrepancies, and to make modifications. Finally, summaries in narrative form were written for each category. Based on these summaries, thematic descriptions were created for the main categories [31]. For the development of the support program, content from categories that pointed out existing needs and requirements was used. The essential information contained in these descriptions is provided in the Results section of this report.

### Phase 2: Objectives, Content, and Strategies

#### Overview

Phase 2 involved defining the content and objectives of the support service, as well as the format and methods with which the support service was to be implemented. The procedure as described in the model of Schneiderhan et al [19] was applied in an adapted form: steps 2 to 4—determining and prioritizing content, writing goals and objectives, and selecting teaching or educational strategies—were combined into one, following the example of Schlutz [20], as the content, objectives, and methods of a learning situation are highly interdependent.

The development of the support program has been guided by Bloom’s [32] taxonomy, which categorizes learning into cognitive, affective, and social domains. To ensure support and acceptability of the support program by the targeted audience and key personnel (eg, nurse managers, leading clinical mentors, and school administration), current conditions, resources, and the results of both empirical studies (survey and focus groups) were also considered during the development of the support program.

#### Data Collection

The goals, objectives, and content were derived from the relevant results of the survey and the focus groups using sequential data integration [26]. The research group met regularly over a period of three months to plan the pilot implementation and testing of the support program. The following data sources were used: results of the web-based survey, results of the focus groups, experts within the institution, and knowledge of internal workflows at the hospital that would facilitate the testing of the support program.

#### Analysis

The research team produced a draft based on a matrix identifying the relevant components of a support program. This structure was linked to the steps of Schlutz [20]. It included information on the theoretical aspects of the support program [19,33], as well as information on practical implementation. Based on the results of the web-based survey and the focus groups, learning objectives were developed, which served as the basis for the content design of the support program. The analyses of the focus groups revealed the participants’ learning needs for several topics. These were prioritized according to their importance and realizability. After that, topics were developed in a creative and iterative group process as frames for the organization of the content of the support program. Topics were operationalized using brief descriptions. At the same time, an educational strategy was defined in terms of format, teaching methods and media, quantity, and timing. Both, topic descriptions and educational strategies, were then entered into the matrix. Experts from the institution were identified and asked to act as potential lecturers at the planned times. Based on the subject areas and descriptions in the manual, they prepared short presentations according to the specified parameters (format, time). The content and methodology were agreed with the members of the research team.

### Phase 3: Expert Validation and Adaptation

#### Overview

This phase included several feedback rounds in which the draft of the support program was presented to various experts from the target group. The feedback from the experts was intended to ensure the feasibility of the support service, as well as to increase the acceptance in the target group.

#### Participants

A total of five experts, all working at a University Medical Center, were consulted. They had the following expertise: adult education in health care, clinical mentoring, practical and theoretical training of nurses, or digitalization and e-learning.

#### Data Collection

The experts were interviewed in a total of four appointments, on three of the four appointments the experts were interviewed individually, on one appointment two clinical mentors were interviewed simultaneously. The interviews were conducted face to face and were structured as follows: first, the experts were presented with the draft of the support service, after which they were able to comment on it openly. Each meeting, and especially the feedback by the experts, was protocolled by a member of the research group.

#### Analysis

The protocols were anonymized; their contents were compiled and then analyzed using qualitative content analysis. Themes and categories resulting from this analysis were discussed within the research group and used for adjustments of the draft produced in phase 2 and finally expanded into a manual with detailed descriptions of all elements for implementing the support program.

### Phase 4: Feasibility Testing (Feasibility and Acceptance)

#### Overview

In phase 4, the support program was implemented according to the manual to assess its feasibility and acceptance. Feasibility in this context refers to the extent to which the intervention can be practically implemented as intended. This includes factors such as the manageability of the program, the availability of necessary resources, and the ability of the target group to use the program as designed [33]. Acceptance was operationalized as participant satisfaction, meaning that the focus was on how well the program was received by users and whether it met their needs and expectations [33].

Prior to the implementation of the support program, experts from the institution were identified and asked to act as potential lecturers at the planned times. Based on the subject areas and descriptions in the manual, they prepared short presentations according to the specified parameters (format, time). The content and methodology were agreed with the members of the research team. A member of the research team was present at each session to provide technical support and moderation. Each short presentation was recorded with the consent of the lecturers so that it could be made available asynchronously at a later date. In order to comply with data protection regulations, the participants’ cameras and microphones were deactivated during the short lecture. After the recording was finished, at the end of the lecture, the participants’ cameras and microphones were activated so that they could take part in the discussion round.

#### Participants

All nursing education providers at the medical center had the opportunity to take part in the support program as part of the feasibility test. Gatekeepers forwarded an invitation to the target group via email with information on the support program. Participation in the support program and the survey at the end of phase 4 was voluntary.

#### Data Collection

A survey design was used to evaluate the support program afterward. A new questionnaire was developed by the research team especially for this study, as no suitable instrument was available in German. The development of the questionnaire was guided by the conceptualizations of Proctor et al [34], Sidani and Epstein [35], and integrated elements of the ABC-SAT questionnaire to assess affective, behavioral, and cognitive domains of satisfaction [36]. The questionnaire was designed as a web-based questionnaire comprising 18 questions on the domains of satisfaction, feasibility, and appropriateness, each of which could be rated using a 5-point Likert scale. Attendance at the relevant events was also requested and there was an option to enter a free text. The demographic information collected in the questionnaire was the participants’ main area of work, age group, and gender. In addition, anonymized data on the number of participants during the sessions and the number of views of the video recordings of the sessions was collected by means of a protocol.

#### Analysis

Numerical data were analyzed descriptively using Microsoft Excel 2016. Textual data (comments of participants) were analyzed using qualitative content analysis.

### Setting

The study was conducted at the University Medical Center Freiburg.

### Ethical Considerations

The conceptualization and implementation of this study were based on the principles of the Declaration of Helsinki. By German law, survey studies with a focus on employees must be approved by the employee council at the respective institution. This body serves to fulfill ethical requirements and safeguard the rights of employees in this country. These responsibilities also refer to the protection of personal rights and data protection in the context of surveys. For this reason, a project description and information materials for potential participants were submitted to the relevant council for approval. The responsible committee of the employee council of the Freiburg University Medical Centre provided approval for this study in written format. Participation in the focus groups was voluntary, no personal data were collected, and anonymity was always maintained. All potential participants received written information on the study (reason for the study, objective, processes, data protection), were informed about the decision of the employee council and its subcommittees, and had the opportunity to contact the investigators in case of questions at any time during the study. Informed consent to participate was assumed if individuals completed the questionnaire and were confirmed (by ticking a box) at the beginning of the questionnaire.

## Results

The presentation of the results of the study follows the structure of the 4 steps of the development process of the support program.

### Phase 1: Needs Assessment

#### Overview

Two focus group sessions with a total of 7 participants (clinical mentors: n=4; nurse educators: n=3) took place in April and May 2022. The first focus group discussion lasted 1 hour and 15 minutes, the second focus group discussion lasted 56 minutes. The characteristics of the participants are provided in Table 1.

**Table 1:** Sample characteristics.

Characteristics			Participants (N=7), n (%)
**Job designation**			
	Clinical mentor	4 (57)	
	Nurse educator	3 (43)	
**Sex**			
	Female	4 (57)	
	Male	3 (43)	
**Age** **(in years)**			
	18-35	2 (29)	
	36-49	3 (43)	
	≥50	2 (29)	

Six main categories emerged from the data, each with several subcategories: (1) existence and use of digital technologies; (2) assessment of (own) digital competencies; (3) support; (4) age, gender, and field of specialization; (5) attitude toward new technologies; and (6) current state of digitization. The last two main categories were derived through an inductive approach from the qualitative data, while the remaining main categories were derived from the interview guide. The development of the support program was based on the analysis of the relevant categories (1), (3), and (6). An overview of these categories and their subcategories is presented in Textbox 1.

**Categorization matrix of the content analysis of the focus groups with relevant content used for the development of the support program.**

**Main category 1: existence and use of digital technologies (deductive category)**

- Existing and used technologies
- Existing and unused technologies
- Desired technologies
- Expectations of technologies

**Main category 2: support (deductive category)**

- Content-related aspects
- Organization and design of support
- Ongoing input and support
- Experiences with existing support

**Main category 3: current state of digitalization (inductive category)**

- Best practice
- Opportunities
- Poor practice
- Barriers

#### Existence and Use of Digital Technologies

This main category covers various aspects related to the use of technology in educational activities. Focus group discussions revealed a wide range of technologies that were already being used, such as digital blackboards, digital learning platforms, laptops, and software for online meetings. However, some existing technologies, like smart boards, were not used, or not used optimally due to a lack of knowledge or partially nonfunctional hardware:

Actually, the other parts [smart boards] are essentially used like whiteboards for writing on. You write on them and then wipe it away. And that approach is not really what one could do.

Focus group 1, nurse educator

Participants expressed a desire for better equipment in the rooms for hybrid teaching, including more laptops, tablets, simulation manikins, and robots. Specific expectations for technologies used in education were also mentioned. Participants want technology to be useful and have a reasonable cost-benefit ratio. Furthermore, it should increase students’ interest and motivation and support the learning process. Additionally, the use of technology should be easy and accessible for collaborative work. The participants also mentioned the benefits of technologies that allow for the easy integration of media into teaching and the documentation of examination or instruction situations.

#### Support

Four subcategories emerged from the data as a result of the discussion about support. This category included participants’ desire for ongoing input and support, their experience with existing support, as well as their ideas, wishes, and needs related to content, organizational, and design aspects of (new) support.

Several participants were already aware of support programs and training on new technologies provided by their institution and had taken advantage of it in individual cases. In this context, even small gains in knowledge were of great importance to the participants. Among other things, access to support programs through a complicated registration process or limited scheduling was viewed critically. Online formats and purely written information were perceived as challenging. The lack of practical exercises was also highlighted as a criticism. In addition, uneven levels of difficulty and limited applicability were named.

It was mentioned that support for nursing education providers during the implementation of new technologies in teaching must be oriented toward different elements, that is, inspiration and support for the correct use of technology in teaching, as well as training in the appropriate use of these technologies. In particular, opportunities for practice were desired. When organizing support, special attention should be paid to interprofessional group formation, different levels of competence, and the requirements of the particular professional groups in regard to new technologies:

I often had the feeling that meanwhile there was a huge gap. Either I get a very marginal introduction ... Or I have something high-end, which is not user-related.

Focus group 1, nurse educator

In addition, participants expressed a desire to contact persons who can provide support in the event of technical problems. Further training should also be programmed on an ongoing basis in addition to regular refresher training. Participants pointed out the challenge of reconciling a busy schedule with training on new technologies.

#### Current State of Digitalization

All participants gave examples and anecdotes of the current state of digitalization and related competencies in the institutions in which they work. In line with the two subcategories, the reports of participants can be divided into “best practice” examples and opportunities, as well as “poor practice” examples and barriers.

In regards to “best practice,” the use of online platforms for communication and information exchange was reported. Other positive aspects mentioned were the training opportunities, internal staff with responsibility for training in certain technologies, and support and mutual collegial support in case of technical problems.

...for example, recently I did a video call into the dialysis unit. And I did the lesson with the colleague via webinar together. Something like that. It all works. That’s the advantage. Thanks to Corona.

Focus group 1, nurse educator

Opportunities for improvement included the need for further development of existing training and high-quality hardware equipment, as well as expanding the monthly work-group meeting to include technical aspects. Participants noted that in order to increase motivation to use new technologies, a recognizable added value must be evident along with comprehensible introductions. In the case of the latter, one participant would like to see better training methods.

Clinical mentors reported examples of poor practice, such as the lack of simulation dolls due to high costs, as well as a lack of training opportunities and responsibilities, and underutilization of existing software despite having the knowledge for an appropriate use. Nursing education providers discussed inadequate use of digital blackboards, limited dates for training, and insufficient differentiation based on competence levels in the training programed. All participants identified common barriers to the use of new technologies, such as high costs, inadequate equipment, lack of time, and lack of practical training space.

Because when I do hybrid teaching with a laptop that is on the table in front, I then occasionally run over and ask if he [a student, note by the authors] can hear and see everything, which of course he cannot...

Focus group 11, nurse educator

Participants also noted individual barriers, such as negative attitudes and lack of experience among colleagues, as well as the challenge of implementing technical solutions into large companies or hospitals due to existing federalist structures. They highlighted the organizational effort of implementing technology, particularly in terms of adapting teaching methods. Additionally, the design and delivery of general IT training from instructors without a background in teaching or nursing was identified as a barrier as the sessions failed to address the needs of the participants.

The study revealed that while a range of tools such as digital blackboards and online platforms are used, some technologies are underused due to limited knowledge or nonfunctional hardware. Participants expressed a need for better equipment, ongoing training, and support, particularly in practical applications of technology and in overcoming barriers such as high costs, inadequate time, and organizational challenges. They emphasized the importance of tailored, accessible support that considers different competence levels and professional needs. Additionally, participants noted both positive and negative experiences with digitalization in their institutions, highlighting the potential for improvement in training and infrastructure.

### Phase 2: Objectives, Content, and Strategies

The following three learning objectives were formulated for the support program, which guided the development process:

- Raising awareness of the topic of digitalization in one’s own field of work
- Promoting motivation to use technologies in nursing education
- Creating opportunities for networking between nursing education providers from different areas of work

In line with Bloom et al [32], participants are expected to develop competencies within the cognitive domain, by gaining essential knowledge about digitalization in nursing education and gaining an understanding of how to apply digital tools to enhance teaching practices. They are also anticipated to cultivate competence within the affective domain by fostering positive attitudes toward digital technologies and increasing their motivation to engage with these tools. Finally, social competence is expected to be strengthened through collaboration and critical engagement with digital solutions, enabling participants to contribute meaningfully to professional discourse on digitalization in nursing education. Based on the objectives, initial topic descriptions were developed for the support program. The draft of the newly developed support program consisted of a series of short online meetings (ie, sessions), which were planned to be programmed once a week over a period of five weeks via the videoconferencing program WebEx, each lasting 15-20 minutes. Each session would focus on one of the topics. The session would start with a topic-centered keynote speech (max 10 min) by a lecturer with expertise in the respective topic followed by a moderated discussion round with the opportunity to ask questions and exchange views on the topic. Every session would be recorded and would later be made available on an institutional digital learning platform. Participants would thus have the option of attending directly or asynchronously. In addition, a forum would be set up on the learning platform to enable participants to interact between sessions.

### Phase 3: Expert Validation and Adaptation

The experts provided feedback on the following aspects of the support program: framework conditions, themes, format, timing, motivation, and potential obstacles. The draft of the support program was generally rated favorably by the experts surveyed. The online format, which does not require registration, the use of software already familiar to the institution, the interactivity of the program (forum and discussion group), and the possibility of addressing questions directly to the relevant experts were highlighted as particularly beneficial features. In particular, the provision of recorded sessions was emphasized, which allows great flexibility for the target group. According to the experts, fixed dates and the short duration of the sessions would enhance motivation and predictability of participation. Experts noted that scheduling the sessions between 1:30 PM and 2 PM would ensure the largest possible number of potential participants could be reached. In addition, experts recommended addressing opportunities for digital collaboration between nurse educators and clinical mentors, some of which are unknown. Based on this feedback, the timing of the program was adjusted accordingly and the topic “Collaboration on a digital learning platform” was added to the lecture series.

Finally, the following five topic descriptions were developed for the support program:

- New nursing technologies at the University Medical Center Freiburg
- Collaboration on a digital learning platform
- Further training programs or further training catalogs for teaching nurses
- Nuts and bolts of Cisco WebEx
- Overview of functions of a locally available online training platform

The manual for the support program was updated accordingly and the anchoring of the support program on the institutional digital teaching-learning platform was finalized.

### Phase 4: Testing (Feasibility and Acceptance)

A total of 25 people enrolled in the course for the support program. Attendance at the four keynote presentations varied, with the second session (collaboration on a digital learning platform) and fourth session (nuts and bolts of Cisco WebEx) being the most attended (n=2), and the third session (educational programs or catalog for teaching nurses) receiving no attendance. The video of the first keynote (new nursing technologies) was viewed most often (n=12), with declining views for every following keynote. With one exception, the testing was conducted as initially planned. The last keynote speech could not be held due to the illness of the lecturer. Overall, the evaluation of the support program involved seven participants, consisting of four male participants, two female participants, and one nonbinary person. Participants included 4 clinical mentors and 2 nurse educators, with one abstention. The median age range was 40-49 years.

Satisfaction with the support program was rated overall as good. Positive responses noted a supportive atmosphere during presentations, high instructor motivation, and well-prepared instructors. However, cognitive feedback indicated less perceived knowledge gain and mixed opinions on content relevance and session length adequacy. Regarding feasibility, participants found the format suitable but noted challenges in integrating it into their daily routines. The digital conference format was generally viewed as appropriate, but the forum and platform usability were deemed less satisfactory. Open-text responses highlighted strengths such as competent speakers and efficient time management, while suggestions for improvement included enhancing educational content relevance and platform usability.

## Discussion

### Principal Findings

In this study, a design-based research methodology was used to develop and test a needs-based support program for nursing education providers to foster digital competencies. The study was conducted in four phases: phase 1 used focus groups to assess the current use of digital technologies and the state of digitalization in nursing education. In phase 2, the objectives, content, and strategies for a support program were developed based on previous survey results and the focus group insights, leading to three main learning objectives: raising awareness of digitalization, motivating the use of technology, and fostering networking among nursing educators. This phase was framed by Bloom et al’s [32] taxonomy, which emphasizes the development of learning in the cognitive, affective, and social domains—each of which is crucial to the development of digital competencies. Phase 3 involved expert validation through multiple interviews, resulting in the refinement of 5 key topics and the finalization of the support program. Finally, phase 4 tested the feasibility and acceptance of the support program using a web-based questionnaire based on the ABC-SAT [36], which showed high satisfaction but low participation and insufficient perceived learning outcomes.

In several countries, including Germany, there are no formal training programs regarding digitalization for nursing education providers [14]. The feasibility test of this support program represents the first step toward the implementation of such a formal training program. This program can be classified as level 1—“reaction” in Kirkpatrick’s model of the “four levels of training” [37]. This means that this program remains rather rudimentary, but has the potential to be expanded and developed further using additional resources. In the absence of a specific guideline for the development of such a support program, the design-based research methodology was used to structure and guide the process of this study. This proved to be extremely helpful, as the steps were comprehensible and the quality of the support program could be improved through iterative and participatory processes. Involving representatives of the target group in the development process brought several advantages: not only did it contribute to increasing the quality of the support program, it also ensured the feasibility of the support program and provided the possibility of easy and low-threshold contact and information for the target group about the planned support program.

The support program aimed to address the entire target group of nursing education providers, which was legitimized with the aim of promoting cooperation between the subgroups of nurse educators and clinical mentors. When designing the content of the support program, a topic-centered approach was taken and an attempt was made to find topics that would appeal to both subgroups of the target group. We chose this approach for several reasons. First, it allowed us to address the immediate interests and needs of both subgroups within our target audience. By focusing on engaging topics, we aimed to maximize initial interest and participation, which is crucial for the success of any educational program. Our goal was to create a stimulating environment where participants would be motivated to actively engage with the content. As a consequence of the topic-oriented approach, it was not possible to design a competence-oriented approach. Although this is not evident from the qualitative and quantitative survey data, there are indications that a competence-oriented design would have been more suitable for the support program. To address this, potential improvements could include introducing competence assessments and providing additional resources while applying a broader variety of learning methods and concepts. This would lead to more needs-oriented programs, especially for the respective target group, and among other things, result in a differentiation of the presented topics into different competence levels (eg, beginner, advanced, and expert) or specific addressing of one of the professional groups. As noted by Sillat et al [38], the increasing number of technologies, frameworks, and strategies underscores the need for assessing and evaluating digital competence in various settings. Given the challenges in self-assessment, which are not limited to nursing settings, the provision of a screening or assessment of digital competencies before choosing a training level should be considered.

The extent to which differentiation into different competence levels would have led to higher participation figures and greater satisfaction with the support program remains to be clarified. With regard to the number of participants, it is noticeable that the recordings of the keynote speeches, which were made available online asynchronously, had higher participation rates than the synchronous online presentations. One possible reason for this could be the stressful and sometimes unpredictable working day of nursing education providers, especially if they work in clinical practice. In addition, the respondents previously reported that they had hardly any time resources, which may be another reason for the low level of participation. This is also confirmed by Dornan [39] stating that employees with a care mandate generally prioritize these tasks higher than participation in continuing education courses. After or during a stressful working day, some individuals may lack the mental capacity to absorb further input in the form of training, as they may already be in cognitive overload [40]. Another reason could be a lack of motivation to participate. In this respect, it is known from adult education that the willingness and motivation to participate in further training depends very much on the general environment of the workplace [41].

These are, for example, the values and mission statement of the organization, as well as the demands and requirements placed on employees in the context of lifelong learning. Learning behavior and thus participation in further training is also strongly dependent on individual learning behavior, learning habits, and personal attitudes [41]. Noteworthy in this context are the different levels of involvement, ranging from enthusiastic to completely absent. While the stakeholders, who also belong to the target group of nursing education providers but tend to work less in direct training and increasingly take on management tasks, were very interested and committed to the development of the support program, there was almost no interest at all on the part of the nurses being involved in direct patient care. We can only speculate about the reason for this great difference in motivation and participation. Since this research project was part of a large, 5-year project with various other research projects, one can also speculate about a kind of fatigue or desensitization to other research projects. Such a phenomenon has already been described by health care professionals [42].

The evaluation recorded only a few participants. However, this number is certainly justifiable and positive in the context of a feasibility test. The evaluation results show an ambivalent picture. In addition to the low number of participants, the results showed little to no increase in knowledge among the participants, yet they stated that they were generally satisfied with the program. A further more in-depth survey of the target group could provide clarity here and answer questions such as which people did or did not take part in the sessions and for what reasons, and whether a different format or a different time would have been more suitable. To address the low participation rate, measures such as enhanced recruitment strategies (eg, engagement of leadership) and flexible participation options (eg, live, asynchronous, and on-demand) should be considered to encourage higher involvement. Additionally, a focus on both intrinsic motivation (eg, emphasizing the value of lifelong learning, team culture, professional identity, and commitment to quality care) and extrinsic motivation (eg, incentives) should be incorporated [43].

### Implications for Practice and Research

Based on the results of this study, several implications for practice can be derived. The next step should be to determine the reasons for participation or nonparticipation in the support program, for example, through a follow-up survey of the target group. This should also address obstacles and facilitating factors.

The support program developed here should also be further developed on the basis of design-based research. When further developing existing support programs or designing new ones, workplace-related factors such as workload, time resources, and working hours should be taken into account. It is also recommended—especially for complex and wide-ranging topics—to pursue a competence-oriented approach in order to best meet the needs of the target group.

### Limitations

This study has several strengths and limitations that should be considered when interpreting the results. First, the sample of this study was relatively small and consisted of individuals from only one medical center. This sample may restrict the generalizability of the findings. However, the evaluation showed an acceptable number of participants for a feasibility study and the issues and findings raised in this study may be still relevant on a national and international level, especially for nursing education and training programs that prioritize the digital competencies of nursing education providers. In this study, the sample was highly specific which improves the credibility [44].

Second, despite a review of existing instruments, no suitable questionnaire could be found, as existing instruments were mostly too generic. Therefore, a self-created questionnaire had to be used for the evaluation, which was based on the ABC-SAT questionnaire [36] and conceptualizations of feasibility and acceptance, but was not validated in its final form.

Third, it is important to be aware of the potential bias of the researchers in this study, as data collection and analysis were conducted by researchers with a background in nursing education [45]. This situation may lead to a better understanding of the context but also affect the interpretation of the data. To compensate for these biases, triangulation was performed during the analysis.

### Conclusions

The research design, consisting of a quantitative survey, a qualitative exploration, and subsequent development with participatory elements of a support program, facilitated both the structured collection of nursing education providers’ views and experiences regarding digital competence and the targeted development of a needs-based support program. However, the likely willingness of the target audience to use the programming in the future appears to be severely limited, as reflected in the low attendance during the synchronous keynote presentations. The reasons for this poor response are varied and may be due to factors such as lack of information or insufficient motivation on the part of the target group. Overall satisfaction was high, but concerns were expressed about the lack of opportunities for professional discourse and individual knowledge gain. Despite these challenges, the overall satisfaction with the pilot implementation suggests that digital and innovative programs have potential and can serve as a basis for further development. Moreover, the insights gained from this study, despite being derived from a localized setting, could be applied to guide the development and implementation of similar support programs in diverse educational and health care contexts.

## Appendix

Multimedia Appendix 1 Key aspects of the training and the characteristics of educating nurses in Germany.

Multimedia Appendix 2 Guidance for reporting intervention development studies in health research (GUIDED) checklist.

Multimedia Appendix 3 Template for Intervention Description and Replication (TIDieR) checklist.

## Abbreviations

GUIDED: Guidance for reporting intervention development studies in health research
TIDieR: Template for Intervention Description and Replication

## Data Availability

The data sets generated during and analyzed during this study are available from the corresponding author upon reasonable request.
