# Supplementary material for: Teaching in the Digital Age—Developing a Support Program for Nursing Education Providers: Design-Based Research
Source: JMIR Form Res. 2025 Jan 15;9:e66109. doi: 10.2196/66109 (PMC11780299; doi:10.2196/66109)
Supplement: Multimedia Appendix 1 [file formative_v9i1e66109_app1.docx]

Key aspects of the training and the characteristics of educating nurses in Germany

In several countries including Germany, nursing is an apprenticeship-based profession. The key aspects of the training are summarized in Table 1. Educational activities during this apprenticeship are performed by nurse educators and clinical mentors. Characteristics of these job designations are depicted in Table 2. Despite the differences in their roles, the terms ‘nurse educator’ and ‘clinical mentor` are referred to as ‘nursing education provider` in this article for ease of reading. However, in cases where it is required, the two job designations are discussed separately in order to highlight the distinctions between them.

Table S1. Vocational training program in Germany.

| **Objects** | **Content** |
| --- | --- |
| Education and qualification/certification | Since 2020: Generalist nursing program resulting in certification as “state certified nurse” |
| Duration and structure | Three years; both theoretical and practical instruction; 10% of practical training hours completed with a clinical mentor |
| Specializations | In third year of training, a geriatric or pediatric nursing specialization is possible |
| Learning areas | Theoretical and practical instruction at nursing schools. Theoretical and practical instruction in different health facilities |

Table S2. Differentiation between nurse educators and clinical mentors.

| **Qualification** | **Nurse Educators** | **Clinical Mentors** |
| --- | --- | --- |
| Certification | Nurses with at least a Bachelor’s degree in nursing education | Nurses with at least one year of professional experience and an additional qualification in preceptorship |
| Location of work | Nursing schools with additional educational visits to students in the practical setting | Healthcare facilities |
| Main responsibilities | Theoretical education and assessment of nursing students | Supervision and mentoring of trainees/students and/or new professionals in their daily clinical work |
| Term for both in this article | **Nursing Education Provider** | |
